# T-Cell lymphoproliferative disorder of hand-mirror cell morphology presenting in an eosinophilic loculated peritoneal effusion, with omental "caking"

**DOI:** 10.1186/1742-6413-3-13

**Published:** 2006-05-08

**Authors:** Richard Siderits, Janusz Godyn, Dearon Tufankjian, Osman Ouattara

**Affiliations:** 1 Laboratory Sciences, Department of Cytology, Robert Wood Johnson University Hospital – Hamilton, Hamilton, USA; 2 Department of Vascular & Interventional Radiology, Robert Wood Johnson University Hospital – Hamilton, Hamilton, USA

## Abstract

**Background:**

Cells with "hand mirror" morphology have not, to the best of our knowledge, been described in a primary effusion sample. This paper describes a case of T-cell lymphoma with eosinophilia in a patient with suspected peritoneal carcinomatosis. Rarely, a T-cell lymphoproliferative process may mimic primary peritoneal carcinomatosis, clinically suggested by a presentation in CT imaging of omental caking with bilateral massive loculated effusions in a patient without lymphadenopathy or splenomegaly.

**Methods:**

A 60 year old caucasian male presented with vague abdominal discomfort and increasing abdominal girth. Computed tomography showed a two centimeter thick omental cake and a small loculated effusion. The clinical presentation and imaging findings were most consistent with peritoneal carcinomatosis. Cytologic evaluation of the effusion was undertaken for diagnostic study.

**Results:**

Rapid intraprocedural interpretation of the effusion sample showed a monomorphic population of cells with "hand-mirror" cell morphology exhibiting cytoplasmic extensions (uropodia) with 3–5 course dark cytoplasmic granules and a rim of vacuolated cytoplasm capping the opposing "mirror head" side. These cells were seen within a background of mature eosinophils. Flow cytometric evaluation of the ascites fluid demonstrated an atypical T-cell population with the following immunophenotype: CD2-, CD3+, CD4-, CD5-, CD7-, CD8+, CD56+. T-cell receptor (TCR) gene rearrangement was positive for clonal TCR-gamma gene rearrangement, supporting the diagnosis of a T-lymphoprolifereative disorder.

**Conclusion:**

A T-cell lymphoproliferative process may present with "hand mirror" morphology in an effusion sample. These cells may show polar cytoplasmic vacuolization and 3–5 course granules within the "handle" of these unique cells. Cytoplasm shows peripheral constriction around the nucleus.

## Background

Cells with "hand mirror" morphology have not, to the best of our knowledge, been described in a primary effusion fluids [1]. The cells were first described and evaluated by EM in 1977 by Shhumacher [2,3]. This paper describes a case of T-cell lymphoma with eosinophilia in a patient with suspected peritoneal carcinomatosis. Malignant effusions can be broadly considered to fall into three main groups 1) obvious population of abnormal cells, dissimilar to mesothelial cells; 2) abnormal cells similar to mesothelial cells; 3) abnormal lymphoid cells within a background of usual mesothelial cells and macrophages [4]. Effusions caused by lymphomas usually involve other body sites before the body cavities [5]. On the other hand primary body cavity lymphoma (primary effusion lymphoma) generally presents as a large B-cell lymphoma involving the pleural, pericardial or peritoneal cavities first. These cases have been associated with HIV infection, immunocompromised transplant recipients or with either herpesvirus-8 (HSV-8) and Epstein-Barr virus [6].

When a suspected lymphoproliferative process is evaluated during an immediate FNA interpretation specimen adequacy evaluation is critical. This can facilitate optimal sample handling for immunohistochemistry, flow cytometry, molecular analysis or chemical/enzymatic evaluation [7].

The present case describes a loculated eosinophilic peritoneal effusion with hand-mirror cells (HMC) in association with peritoneal caking. Clinical suspicion based on CT imaging included peritoneal carcinomatosis. This ascites fluid also showed an increased relative proportion of unremarkable eosinophils. Eosinophilic effusions (defined as exceeding 5% eosinophils) have been associated with many etiologies including trauma, infection, peritoneal dialysis and malignancy [8]. Both the ascities and omental caking described in this case may be seen with lymphoproliferative processes. For example peripheral T-Cell lymphomas can rarely present as ascites [9]. Hodgkin's lymphoma, Burkitt's lymphoma, HIV associated lymphomas, blastic (NK)-cell and Ki-1 lymphomas have also been associated in the literature with omental caking and accompanying ascites [10-14].

## Methods

This 60 year old Caucasian male presented to his primary care physician with a chief complaint of vague abdominal pain and increasing abdominal girth of 3 months duration. His past medical history was essentially unremarkable.

Peripheral blood showed mildly elevated WBC and platelet counts with mild relative lymphocytopenia and normal eosinophil counts. Carcinoembryonic antigen, Alpha-feto protein, CA15-3 and CA125 were all within normal ranges. Calcium was consistently mildly decreased during the course of hospital stay with slightly elevated prothrombin times.

**Figure 1:**
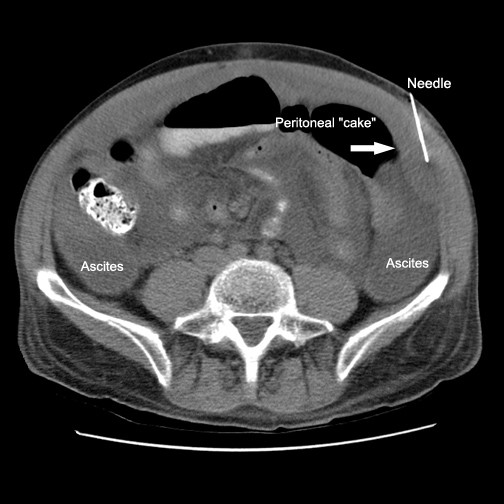
CT image showing loculated ascites, FNA needle and omental/peritoneal "cake".

Initial imaging of abdomen by computed tomography (CT) demonstrated large volume ascites, fatty changes of liver, and omental-peritoneal "caking" measuring up to 2.0 cm in thickness (Figure: 1). There was no enlargement of pelvic/peritoneal nodes or spleen. The prostate appeared unremarkable.

Ultrasound guided aspiration of ascites fluid was obtained for routine cytology using a 22 gauge needle. Following the initial ultrasound guided paracentesis a CT guided Fine needle aspirate samples of omental cake was performed using an 18 gauge core biopsy. Initial aspirate samples were evaluated onsite for specimen adequacy by the cytopathologist. Additional samples were retained for ThinPrep, cell block, core Bx and microbiologic culture. The patient tolerated the procedure without difficulty. Rapid interpretation documented a sample adequate for cytomorphologic evaluation, special studies and microbiologic culture.

## Results

**Figure 2:**
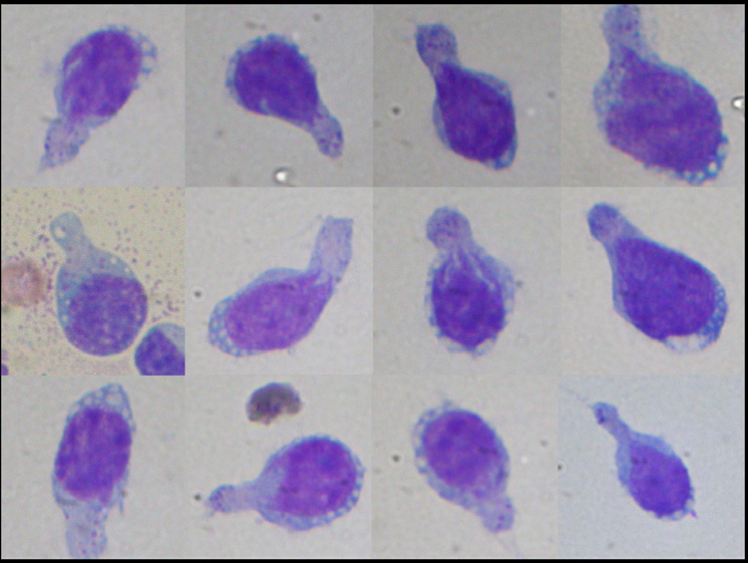
"Hand-mirror" cells with uropodia, vacuolated cytoplasm near nuclear rim and scattered course cytoplasmic granules within uropod (Oil).

**Figure 3:**
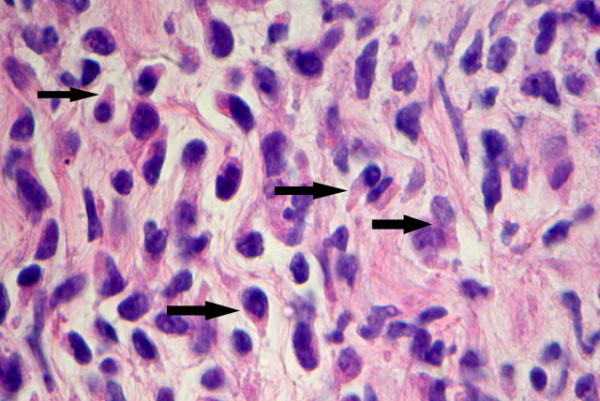
Histologic features from FNA micro-biopsy sample showing frequent cells with Hand-Mirror morphology. Other areas in this tissue and in effusion sample showed increased proportion of eosinophils and plasma cells (H&E stain, 40 × magnification).

Cytologic interpretation of the FNA for both the left flank omental mass and ascites specimen showed an atypical lymphoid population with prominent hand-mirror cell morphology and frequent eosinophils. This was felt to be strongly suspicious for a lymphoproliferative process, possibly T-Cell derived (Figure: 2). The nuclei of these cells showed a smooth contour with irregularly clumped dark chromatin. Nuclear protrusions were occasionally noted extending toward the handle portion of the hand mirror cells. Nucleoli were prominent, basophilic in diff-Quik stained smears and often multiple. Mitoses, cleaved nuclei, nuclear granularity and apoptotic cells were infrequent. Histologic features seen in the subsequent core biopsy displayed surprisingly well preserved HMC differentiation and showed a monomorphic population of hand-mirror cells within a background of mature eosinophils (Figure: 3).

Flow cytometric and Immunohistochemistry revealed an atypical CD56+ T-Cell population suggestive of a T-Cell lymphoproliferative process. PCR analysis for TCR gamma gene rearrangement revealed two monoclonal peaks with TCR gamma primers, corresponding to V-gamma 11 gene rearrangement and V-gamma [1] gene rearrangement. Both EBV (EBER) for early RNA by ISH and Herpesvirus-8 were negative. These findings collectively supported the final diagnosis of a T-cell lymphoma with predominant Hand Mirror Cell differentiation.

## Discussion

This was a case of a 60 year old man with loculated peritoneal eosinophilic effusion and omental caking who was diagnosed with T-cell lymphoma by FNA. Cytologically distinct hand-mirror forms comprised the majority of the cells in the effusion. The finding of hand mirror cells in a peritoneal effusion has not, to the best of our knowledge, been described in the current literature. The patient declined treatments, entered hospice care and died within three months of the diagnosis.

The term hand-mirror cell differentiation is descriptive. The cell is characterized by an asymmetrical cytoplasmic elongation extending out from one pole of the nucleus (uropod). Both the nucleus and the elongated cytoplasmic "handle" together give the appearance of a hand-mirror. The hand-mirror cell morphology had in the past been classified as a distinct morphologic variant of acute lymphoblastic leukemia [15]; however, the natural history of this subtype of the disease did not warrant keeping this as a separate entity in the classification. These cells are proportionately dominant in less than 1% of ALL and are not associated with any particular immunophenotype or distinct prognosis [16].

Hand-mirror cells have also been reported with many diagnostic entities including cutaneous natural killer cell lymphomas [13], Anaplastic Ki-1 lymphomas [14], acute lymphoblastic leukemia [15], Burkitt's lymphoma [17], other B-Cell lymphomas with surface IgM-lambda [18], and with granulocytic and acute myelomonocytic leukemia [21]. This morphology can also be seen in non-neoplastic conditions, such as meningitis [22]. An animal model has been defined for study of the hand-mirror cell phenomenon [19].

The distinct morphology most likely represents an aspect of immune responsivity or challenge involving the incorporation of immune complexes[22]. Although an easily recognized morphologic form, the hand mirror cell morphology has generally been considered to be of limited prognostic value [16] with the exception that the recognition of this morphology points to a lymphoid lineage.

The CT findings in this case were associated with peritoneal involvement by lymphomatosis however the CT observation of omental "caking" has been identified as an unreliable means to distinguish peritoneal carcinomatosis or tuberculous peritonitis from lymphomatosis. Ascites without loculation or septation and diffuse distribution of enlarged lymph nodes may however help to differentiate peritoneal lymphomatosis from other eitiologies [24].

## Conclusion

This case also illustrates advantages of on-site specimen adequacy evaluation which proved essential in obtaining samples for appropriate special studies.

Immunophenotypic, flow cytometric and molecular studies in this case documented a T-Cell lymphoma. It also provided an opportunity to offer a rapid interpretation which in this case did not support the provisional diagnosis of peritoneal carcinomatosis which had been based on CT observation of omental caking. The preponderance of hand mirror cells and the presence of an eosionophilia in the effusion were consistent with the final diagnosis.
